# Advances in Antiwolbachial Drug Discovery for Treatment of Parasitic Filarial Worm Infections

**DOI:** 10.3390/tropicalmed4030108

**Published:** 2019-07-18

**Authors:** Malina A. Bakowski, Case W. McNamara

**Affiliations:** Calibr at Scripps Research, La Jolla, CA 92037, USA

**Keywords:** *Wolbachia*, filaria, parasitic worms, *Onchocerca*, *Brugia*, drug discovery, antiwolbachial, endosymbiont, neglected tropical disease, high-throughput screening

## Abstract

The intracellular bacteria now known as *Wolbachia* were first described in filarial worms in the 1970s, but the idea of *Wolbachia* being used as a macrofilaricidal target did not gain wide attention until the early 2000s, with research in filariae suggesting the requirement of worms for the endosymbiont. This new-found interest prompted the eventual organization of the Anti-*Wolbachia* Consortium (A-WOL) at the Liverpool School of Tropical Medicine, who, among others have been active in the field of antiwolbachial drug discovery to treat filarial infections. Clinical proof of concept studies using doxycycline demonstrated the utility of the antiwolbachial therapy, but efficacious treatments were of long duration and not safe for all infected. With the advance of robotics, automation, and high-speed computing, the search for superior antiwolbachials shifted away from smaller studies with a select number of antibiotics to high-throughput screening approaches, centered largely around cell-based phenotypic screens due to the rather limited knowledge about, and tools available to manipulate, this bacterium. A concomitant effort was put towards developing validation approaches and in vivo models supporting drug discovery efforts. In this review, we summarize the strategies behind and outcomes of recent large phenotypic screens published within the last 5 years, hit compound validation approaches and promising candidates with profiles superior to doxycycline, including ones positioned to advance into clinical trials for treatment of filarial worm infections.

## 1. Introduction

Filarial parasitic worm infections afflict millions of people worldwide. These diseases are not commonly lethal, but the associated morbidities can cause significant physical, psychological and economic suffering for infected individuals, families, and communities. The prolonged chronic nature of infections and propensity for causing irreversible damage further compounds the negative impact of these diseases. Filarial worms of greatest concern to global health include *Onchocerca volvulus*, the causative agent of onchocerciasis (river blindness), and *Wuchereria bancrofti*, *Brugia malayi* and *Brugia timori* that are the causative agents of lymphatic filariasis (elephantiasis) (listed in Table 1 with other species relevant to the antiwolbachial approach, either as targets or model organisms). Most *O. volvulus* infections occur in sub-Saharan Africa, with small foci of infection occurring in South America and Yemen. Altogether, 205 million people live in areas where they are at risk of becoming infected and about half a million are estimated to be blinded by the disease. Lymphatic filariasis is endemic in an even greater geographic area, found in the tropics of Asia, Africa, the Pacific, and Americas, with 886 million people at risk of infection and 40 million with disfiguring and disabling physical symptoms.

With many afflicted, and even more individuals at risk of developing infections, global programs to eliminate filarial infections have been ongoing for a long period of time, from vector elimination programs initiated in 1975 to mass drug administration campaigns (MDA) with drugs that halt transmission of the infections [24]. However, significant challenges stand in the way of elimination goals, not the least of which is the lack of drugs that can effectively eliminate adult worms and cure the infections. The discovery of a bacterial endosymbiont, *Wolbachia*, required for viability and fecundity of adult worms causing both diseases, has provided an opportune target for drug discovery efforts. Proof-of-concept studies demonstrated the viability of this approach in human patients treated with the antibiotic doxycycline for 4 to 6 weeks, a treatment time that is unfortunately prohibitive for MDA and limited by contraindications for children and during pregnancy. Therefore, drug discovery efforts have concentrated on developing antiwolbachial therapies that are safer and effective even when given in abbreviated dosing schedules, allowing distribution to all in need of treatment. Here we detail the rationale, challenges, strategies and recent progress in developing novel antiwolbachial therapies.

## 2. Filarial Worm Infection and the Antiwolbachial Approach

### 2.1. Filarial Worm Infection Biology—An Overview

Worms that cause onchocerciasis and lymphatic filariasis are transmitted via the bites of an insect vector. In the case of onchocerciasis, *O. volvulus* are transmitted by black flies of the *Simulium* spp. that breed in fast-flowing waters of rivers. During a blood meal, infectious larvae exit the fly head and migrate into the skin of the host. Once inside the human host, the larvae develop into adults, called macrofilariae. Filarial worms have extraordinary lifespans, with *O. volvulus* worms estimated to live up to 14 years inside the human host [25]. In that time, the large female worms (up to 50 cm long) reside in subcutaneous nodules and release progeny called microfilariae, estimated at 16,000 per day [26], that migrate through the skin and eyes of the infected, with smaller males known to travel between nodules to fertilize the females. Black flies take up the migrating microfilariae during feeding, and these develop inside the fly into infectious larvae that can go on to infect new hosts. In addition to the nodules, symptoms of infection are associated with inflammation due to large numbers of microfilariae that migrate and die in the skin for many years. This includes intense itching and skin disease, and in extreme cases, visual impairment and even blindness. Neurological disease has also been reported.

Worms that cause lymphatic filariasis are transmitted by several mosquito species. Inside the human host the larvae develop into adults in the lymphatic system, not in subcutaneous nodules, and microfilariae shed by the macrofilariae circulate in peripheral blood, where they can be ingested by mosquitoes during a blood meal. Worms that cause lymphatic filariasis are smaller than *O. volvulus* (females are 4.3 to 10 cm long depending on the species) but like *O. volvulus* can live for long periods of time (up to 8 years [25]) during which they continue to shed large numbers of microfilariae. Many such infections are asymptomatic, but nonetheless cause damage to the lymphatics and kidneys. The visible symptoms of infection include swelling (lymphedema) of extremities and genitals (hydroceles in men), fevers, inflammation, secondary bacterial and fungal infections and acute dermato-lymphangio-adenitis attacks [27]. As the disease progresses, patients may develop elephantiasis, marked by gross swelling, hardening and thickening of the skin.

### 2.2. Approved Therapies and Accompanying Challenges

Current treatments against filarial worms (ivermectin, diethylcarbamazine (DEC), albendazole) (Table 2), alone or in combination (Table 3), are only partially effective against the long-lived macrofilariae. While the combination of DEC and albendazole has been reported to exhibit significant macrofilaricidal activity against worms that cause lymphatic filariasis [28,29] the main effect of the treatments is to temporarily sterilize the adults. Thus, with additional microfilaricidal activity of ivermectin and DEC, they serve as important transmission-blocking preventive chemotherapies. In the case of onchocerciasis, the treatment can also alleviate symptoms brought on by the numerous progeny that circulate or migrate through the body of the infected human host and that are mostly responsible for clinical manifestations of this disease. MDA campaigns, where treatments are administered annually or bi-annually for many years (due to long reproductive lifespan of filariae) have been successfully employed to reduce disease burden and in some cases even eliminate transmission of filarial worm infections from certain regions. This includes the successful elimination of transmission of onchocerciasis from most of Central and South America with MDAs supported by the Onchocerciasis Elimination Program in the Americas (OEPA) [30,31] and in Western Africa, where vector control and MDA were undertaken with the support of the Onchocerciasis Control Programme (OCP) [32]. These are great global health and philanthropic (donations of Mectizan (brand name of ivermectin) by Merck, albendazole by GSK, and DEC by Eisai) success stories. However, despite tremendous progress due to sustained MDA efforts over nearly 25 years [33], significant barriers still exist in achieving elimination goals for these widespread and disabling neglected tropical diseases.

Sustained MDA coverage is complicated by geo-political issues, patient compliance (e.g., due to fear of adverse reactions to treatment), but also by the lack of macrofilaricidal activity of the drugs themselves, which requires repeated annual or bi-annual dosing for the reproductive life-span of the adult filariae (9–11 years in the case of onchocerciasis). Special barriers to treatment are also present in areas co-endemic for *Loa loa* (West and Central Africa), as patients infected with *L. loa* can develop adverse reactions following administration of microfilaricidal therapy. The adverse reactions can range between mild, moderate, to serious non-neurological and neurological reactions [34]. The neurological severe adverse events (SAEs) can cause loss of consciousness, can be fatal and are associated with high titers of *L. loa* microfilariae in the blood (>30,000 microfilariae/mL). Therefore, treatments for onchocerciasis in *L. loa* co-endemic areas are not recommended and in the case of LF, albendazole monotherapy may be applied (Table 3); however, the overall antifilarial effectiveness (microfilariae prevalence measured over 2 weeks to 12 months after treatment) of albendazole has been questioned [35], and other approaches are being investigated. For example, the Test and Not Treat (TnT) method, enabled by development of a mobile phone microscope, advocates for identification of patients who should not receive microfilaricidal treatment due to high burdens of circulating *L. loa* microfilariae and the associated risk of developing SAEs. Additionally, development of an effective and safe (e.g., does not kill *L. loa* microfilariae) macrofilaricidal therapy that can be widely administered in resource limited settings would undeniably advance elimination of infections causing onchocerciasis (and lymphatic filariasis) in all endemic areas (Box 1).

Box 1 **An abridged, working macrofilaricidal target product profile (TPP) for onchocerciasis from the Macrofilaricide Expert Group (MEG) and Macrofilaricide Drug Accelerator (MacDA) Program.**

**Effective**

- Results in death or permanent sterilization of adult onchocerciasis worms (*O. volvulus*).
- After one course, death or permanent sterilization of adult worms is achieved (minimum in 70%; ideally in 100%).
- Oral dose, once daily, up to 7 days or a single, intra-muscular injection.

**Safe**

- Target Population: at minimum, all individuals ≥ 5 who are infected, excluding pregnant women; ideally all individuals at risk for onchocerciasis.
- Safe for use in patients co-infected with *L. loa* (i.e., no rapid microfilaricidal activity).
- No significant drug–drug interactions.
- No monitoring for adverse events or monitoring manageable at local healthcare posts.

**Amenable for use in resource limited settings**

- Able to be delivered by health care facility or ideally by an appropriately trained community volunteer.
- Stable for ≥ 3 years in Zone 4B (30 ± 2 °C, 75 ± 5% relative humidity).
- At minimum, ≤ $2.00 per course of therapy; ideally ≤ $0.30.

### 2.3. Wolbachia, an Attractive Anti-Macrofilarial Target

*Wolbachia* is a genus of gram-negative obligate intracellular bacteria related to *Rickettsia*. First noted in *Culex pipiens* mosquitoes [10] *Wolbachia* have subsequently been found to infect numerous invertebrate species including insects (40–65% of insects have been estimated to be infected) and filarial nematodes [36,37,38]. In insects, *Wolbachia* are mostly maternally inherited sexual parasites known to manipulate host reproduction through cytoplasmic incompatibility, feminization and male killing, thereby increasing the proportion of infected hosts in the population. Evidence for horizontal transfer between hosts is also recognized. *Wolbachia* infections in mosquitoes have been observed to reduce brood size, shorten lifespans of adults, and reduce transmission of malaria, dengue, Zika and chikungunya, and are thus being exploited to control the spread of these diseases [39]. In contrast, in infected filarial nematode species *Wolbachia* is a maternally inherited endosymbiont required by the worms for their viability and reproduction (Table 1 and Table 4). 

*Wolbachia* have a somewhat controversial [52,53] taxonomy, with all bacteria currently assigned to the same species, *Wolbachia pipientis,* but classified as belonging to distinct supergroups based on phylogenetic relationships derived from sequence analysis of ribosomal, *ftsZ*, and *wsp* genes. The supergroups are further divided into strains. Despite being classified as a single species, comparative genome sequencing studies have revealed that *Wolbachia* genomes can be highly rearranged, with many breaks in synteny and flexible gene assortments between supergroups [54]. The differing associations with their hosts (sexual parasite in insects versus obligate mutualist in filariae) are also reflected in *Wolbachia* genomes. Endosymbionts of *Onchocerca* spp. of worms and that of the dog heartworm *Dirofilaria immitis* belong to supergroup D and have the smallest genomes out of the currently sequenced filarial *Wolbachia*, just under 1 megabase (Mb) in size, and encoding for less than a thousand proteins (Table 4). The *Wolbachia* of *W. bancrofti*, *Brugia* spp. and that utilized in rodent efficacy models, *Litomosoides sigmodontis*, belong to supergroup C, and currently published genomes of this supergroup have similarly reduced genomes, approaching 1.1 Mb in size. *Wolbachia* strains that infect fruit flies (*Drosophila melanogaster*) and mosquitoes (*Aedes albopictus*) used in drug discovery high-throughput screening efforts belong to supergroups A and B, respectively. Their genomes are approximately 1.3–1.5 Mb in size with a larger assortment of protein coding genes (1100 and 1205, respectively), potentially reflecting the slightly more parasitic rather than mutualistic relationship with their hosts (Table 5) [50]. This still represents a marked genome reduction and gene loss compared to free-living bacteria (e.g., the *E. coli* K-12 genome of 4.6 Mb encodes for nearly 4300 protein coding genes). However, the extreme genome reduction is not an unusual feature of *Wolbachia*, as other host-dependent obligate intracellular parasites like bacterial *Chlamydia* spp. [55] and *Rickettsia* spp. [56], and the eukaryotic pathogens microsporidia [57] that rely on the host for nutrients and a replicative environment all have highly reduced genomes. This genome reduction has been linked to the adaptation of these organisms to an obligate intracellular lifestyle and strong dependence on the host: with some metabolic functions provided by host cells, the parasites may dispense with genes involved in biosynthesis or regulatory pathways.

Despite their reduced genomes, *Wolbachia* have a large impact on their filarial hosts. Initial experiments showed that tetracycline treatments, used for outbreaks of staphylococcal dermatitis in jirds housed in animal facilities, could prophylactically prevent infection by *B. pahangi* filarial nematodes [58]. The finding that tetracycline treatment affects development and fertility of *Wolbachia*-containing *L. sigmodontis in vivo* but *Wolbachia*-free species are not affected by tetracycline treatment spurred further investigations into the relationship of *Wolbachia* with their filarial hosts and the potential for the bacteria to be used as targets for developing antifilarial therapies [59]. *Wolbachia* reside in the lateral hypodermal chords of filarial nematodes and in the ovaries and developing embryos within female worms. Elimination of *Wolbachia* with antibiotics causes sterility (an “embryostatic” effect) of female worms and slow death of the adult filariae. In general, the sterilizing effect has been attributed to widespread apoptosis immediately following *Wolbachia* elimination in the adult germline, the somatic cells of embryos and the microfilariae [60]. More recently, *Wolbachia* has been shown to be required to maintain proper development of filarial germline stem cells and for the stimulation of mitotic proliferation in the ovary [61].

Precisely why *Wolbachia* is required by filariae is a matter of debate and has been discussed in detail elsewhere [62]. Regardless of the precise mechanism, it has been shown in human clinical trials that doxycycline therapy at 200 mg/kg given over 4 to 6 weeks has sterilizing and macrofilaricidal effects on *O. volvulus* [63,64], the causative agent of onchocerciasis. Sustained sterility of adult worms has clear applications for transmission blocking. Additionally, the death of *O. volvulus* macrofilariae occurred over a period of 1 to 2 years, and this gradual demise of large adult worms is considered a benefit of antiwolbachial therapy, as this, together with elimination of *Wolbachia* itself, may limit development of inflammation. Similar results were observed in human trials looking at efficacy of doxycycline treatments on lymphatic filariasis [19,65,66,67,68], where antiwolbachial treatments had sterilizing and macrofilaricidal effects, and via meta-analysis and other trials involving *O. volvulus* [69,70]. The antiwolbachial approach has also been applied to the treatment of heartworm infections in dogs, where doxycycline is part of the ideal treatment regimen. However, the long duration of doxycycline therapy in onchocerciasis and lymphatic filariasis makes it a challenge to administer. Moreover, doxycycline is contraindicated in children ≤ 8 years of age and in pregnant women. Thus, a concerted effort has been undertaken to identify shorter, safer and more effective antiwolbachial therapies for the treatment of filarial infections. We next summarize the approaches to and findings of these antiwolbachial drug discovery efforts.

## 3. Antiwolbachial Drug Discovery

### 3.1. Phenotypic Antiwolbachial High-Throughput Screening

As obligate intracellular bacteria, *Wolbachia* must be cultured inside of its host’s cells. As no nematode cell lines have been developed, researchers have turned to using insect cells hospitable for *Wolbachia* replication and amenable to growth in vitro in order to identify antiwolbachial compounds in phenotypic screens (Table 5). JW18 [71] and LDW1 [72] are cell lines derived from *D. melanogaster* flies infected with *Wolbachia* strain *w*Mel that can be repeatedly passaged without loss of the bacteria. These lines express a fluorescent marker, the Jupiter-green fluorescent protein (GFP) fusion protein, that associates with microtubules [73]. The LDW1 cell line also expresses histone-RFP fusion localized to the nucleus of the cells [74]. The GFP and RFP tagged proteins make these lines especially attractive for high content image-based screening that relies on identification of *Wolbachia* using DNA staining and for cellular microbiology investigations [71,72,75,76]. Mosquito cell lines have also been used for *Wolbachia* drug discovery. One of the first insect cell lines used for antiwolbachial drug profiling, the *Ae. albopictus* cell line Aa23, is naturally infected with *w*Alb strain of *Wolbachia* [77]. The *w*Alb strain has also been transferred from Aa23 cells to the *Ae. albopictus* C6/36 cell line and used extensively in high-throughput screens (Table 5).

A preferred detection method for high-throughput screening of chemical libraries containing thousands to hundreds of thousands of compounds is specific and relevant, inexpensive, simple, and compatible with automated liquid and plate handling equipment. To minimize costs, reduce compound consumption and increase throughput it is often desirable to miniaturize assays so that they produce good quality data providing clear distinction between positive and neutral controls (i.e., a sufficiently high signal-to-noise ratio) while using significantly less materials, e.g., by optimizing assays to be run in the lower volume 1536-well plates instead of in 384-well plates. This represents an approximate four-fold increase in throughput and proportional reductions in cost. No fluorescently tagged *Wolbachia* strains have been created, as no genetic tools to manipulate these bacteria have been developed, thus detection of *Wolbachia* within host cells in a manner amenable to HTS and miniaturization is not trivial. However, to visualize and quantify *Wolbachia* within host cells several approaches have been employed, including Giemsa staining [80], real-time quantitative PCR (qPCR) [77], quantitative reverse transcription PCR (qRT-PCR) [92], fluorescent nucleic acid staining (e.g., propidium iodide, 4′,6-diamidino-2-phenylindole (DAPI), and SYTO 11) [94,95], *Wolbachia*-specific antibody staining [77,96] and 16S rRNA fluorescent in situ hybridization (FISH) [76,97,98] (Table 6). 

All of the above *Wolbachia* detection methods except for Giemsa, PI staining and qRT-PCR have been applied to antiwolbachial high-throughput screening, including non-specific (fluorescent nucleic acid stains) and *Wolbachia*-specific (qPCR, antibodies and 16S rRNA FISH) approaches (Table 6). For example, using qPCR in 96-well plate format, 2664 approved drugs, bioactive compounds and natural products (CRX; CombinatoRx, Singapore, Singapore) and 10,000 BioFocus compounds were screened in C6/36 cells to identify repurposing opportunities [88] and six structurally diverse chemotypes with in vitro antiwolbachial activity [87]. Using the JW18 cell line and DAPI staining of *Wolbachia* and host DNA, a high-content imaging screen of nearly 5000 compounds screened in 384-well plates with an ImageXpress Micro (Molecular Devices, Sunnyvale, CA, USA) automated imaging system equipped with a 40× objective (10 fields per well imaged) identified 40 putative hits as having antiwolbachial activity [71]. SYTO 11 staining of live C6/36 cells in 384-well plate format, imaged using a confocal 60× objective of an Operetta automated imaging system (3 images per field, 5 fields per well imaged) was also successfully used to screen for antiwolbachial compounds [89] and determine potency of otherwise identified hits [87]. 

While the DAPI and SYTO 11-based methods produced functional high-content imaging screens, the unspecific nature of these stains requires the use of high magnification objectives to discriminate effectively between *Wolbachia* and host DNA-derived signals, and the acquisition of multiple fields of view per well to achieve acceptable assay quality, limiting assay throughput. To overcome this limitation, *Wolbachia*-specific staining has since then been applied in high-content imaging assays. For example, an antibody raised against the peptidoglycan-associated lipoprotein from *B. malayi* (*w*BmPAL) was used to stain C6/36 infected cells in 384-well plate format to screen AstraZeneca’s entire 1.3 million compound library, identifying 9 potential hit series [90]. While the approach relied on a specific limited reagent (anti-*w*BmPAL), the signals from plates were analyzed relatively quickly with a fluorescent microplate cytometer (TTP acumen Explorer eX3, Melbourn, UK) paired with Hoechst staining and a plate reader to detect host cell density (Perkin Elmer EnVision, Waltham, MA, USA), increasing throughput over previous high-content imaging approaches. Finally, 16S rRNA FISH staining optimized for high-throughput screening, which uses inexpensive and easily customizable fluorescently-labeled oligonucleotides and also results in a *Wolbachia*-specific signal, allowed us to screen over 300,000 compounds from Calibr’s focused diversity and bioactive libraries, identifying 288 potent and selective hits, including known drugs and novel chemical entities such as the quinazoline heterocycles [92]. This was initially done in 384-well plate format, and upon further miniaturization in 1536-well plate format using a CX5 CellInsight Cellomics high-content imaging instrument (Thermo Fisher Scientific, Waltham, MA, United States) with a 10× magnification objective, requiring only a single field of view per well, thereby significantly increasing throughput.

### 3.2. Validation of Antiwolbachial Compounds Identified in Vitro Screens

The large phenotypic screening efforts outlined above have yielded much novel chemical matter with potential antiwolbachial activity. More obvious in vitro hits, such as analogs of already validated antibiotics, have been successfully advanced for in vivo proof-of-concept testing (e.g., minocycline, a derivative of doxycycline [99]). Analysis of chemical structures to censor compounds that are obviously intractable for development is a standard procedure in drug discovery and serves to narrow the focus to candidates without clear liabilities. However, validation of the remaining promising in vitro hits against filarial *Wolbachia* continues to represent a bottleneck in the antiwolbachial discovery process. As discussed above, there are marked differences between *Wolbachia* genomes and lifestyles of strains from insect and filarial nematodes, and it is possible that compounds identified in insect-cell screens will have strain-specific activity. The phenotypic screening approach and reliance of *Wolbachia* on host cells for survival and a replicative environment also makes it possible that compounds identified will have host-specific activity. And, fully-developed adult worms, the target of antiwolbachial therapy, present a significant permeability barrier, and may block entry, metabolize, or excrete active compounds. Many “straight-off-the-screening-deck” compounds have also not been chemically optimized to maintain proper exposures and metabolic stability in in vivo models. It is no surprise therefore, that many of the promising potent antiwolbachial compounds identified in insect cell-based screens show disappointing activity against *Wolbachia* in filarial nematodes in vitro or in vivo. 

Another layer of complexity is that the macrofilaricidal effect on adult worms following elimination of *Wolbachia* takes a long time to be observed, 1–2 years in the case of *O. volvulus* in human patients [63]. Thus, the primary readout of validated compounds even in in vivo models is usually elimination of *Wolbachia*, which, if insufficient, is reversible, as is the embryostatic effect it imparts. It is unclear what is the precise threshold of elimination required for a sustained effect (estimated to be >90% in patients based on doxycycline clinical trials [4]), but incomplete elimination of the bacteria can lead to eventual repopulation of worm tissues (e.g., as seen with a 14-day 40 mg/kg *bid* (*bis in die*, twice a day) doxycycline treatment in the jird/*L. sigmodontis* model of infection [86]). Moreover, the specific elimination thresholds that must be achieved in disparate preclinical models to translate to clinical efficacy are also topics of deliberation, with clear answers likely to emerge as more compounds vetted in these systems enter clinical trials.

Not counting the bovine *O. ochengi* model [3], the most reliable and physiologically relevant validation tools are the in vivo rodent filarial infection models that have been developed to study filarial diseases and where sustained *Wolbachia* elimination, and in some cases even the embryostatic effects, can be observed following compound treatment [4,17,45,92,100]. However, these models require large amounts of chemical matter, can take weeks to months in order to establish and perform, and compounds not optimized to achieve high enough exposures in rodents may give needlessly discouraging results. In addition to the significant time and resources required, the ethical challenges presented by excessive use of such models as secondary validation screens are also an obstacle: a typical high-throughput screen may identify hundreds of attractive hits. The timelines involved, also preclude rapid turnaround for efficient medicinal chemistry optimization cycles. Therefore, other faster and less-resource intensive methods of validating activity of compounds against filarial *Wolbachia* have been developed and employed.

Validation of antiwolbachial compounds in filarial nematodes outside of in vivo models faces unique challenges. *Wolbachia* distribution between different developmental stages and even different worms of the same sex and age is variable. Additionally, without more complicated assay systems such as provision of feeder cell monolayers [40,101], parasitic worms can be maintained outside of their host (ex vivo) for limited amounts of time. Nonetheless, validation against filarial *Wolbachia ex vivo* has been performed using microfilariae and adult male and female worms, using several quantification methods and filarial species. 

The most abundant filarial life stage are the microfilariae, which are easily accessible in large quantities from infected animals and may serve as surrogates for adult filariae. However, microfilariae contain significantly less *Wolbachia* then adult worms, do not have developed reproductive systems where *Wolbachia* resides, and are arguably much more permeable and susceptible to compound treatments. Male worms have also been used to validate antiwolbachial compound activities, as they are smaller than females and more convenient to work with (e.g., in a sister species of the human parasite *O. volvulus* that infects cattle, *Onchocerca ochengi* [3], adult females are 25 cm and males are 3 cm long [43]). Yet, the males may also represent a lower threshold for compound validation as they do not contain *Wolbachia* in their reproductive tracts, and due to this fact and the relative size difference contain significantly less *Wolbachia* compared to females. Importantly, clearance of *Wolbachia* from all tissues, including the reproductive tract, may be important for compound efficacy in vivo, and long-term sterility and eventual death of the filariae. For example, *Wolbachia* in the ovaries of *O. ochengi* are more resistant to rifampin antibiotic treatment in cattle than *Wolbachia* in hypodermal chords of either male or female worms [102] and this population, if not eliminated, may eventually recover in the female reproductive tract following cessation of treatment. Similarly, we observed that the *Wolbachia* population found in the reproductive tract of *B. pahangi* adult female worms is more resistant than the population found in the hypodermis to an ex vivo treatment with doxycycline and a novel series of antiwolbachial quinazolines, as analyzed through imaging of *Wolbachia* in ovaries with 16S rRNA FISH and qRT-PCR performed on whole worms and specific worm tissues [92]. Conversely, immunohistochemical observations in *D. immitis* following doxycycline treatment suggested complete clearance of *Wolbachia* from ovaries of worms and a marked decrease in number of *Wolbachia* in the hypodermis [96]. These observations are difficult to reconcile with the available data, but their incongruity may be due to the timing of treatments and observations. While it has been established that during development *Wolbachia* from the hypodermis infects the female reproductive tract [98,103], the replication of *Wolbachia* within the female gonad and the dynamics of *Wolbachia* populations within worms in response to specific compound treatments are yet to be defined. In fact, it is possible that nearly complete elimination of *Wolbachia* from the hypodermis is needed for efficacy, and it is correlated to elimination in the ovaries in the above examples.

**Figure 1:**
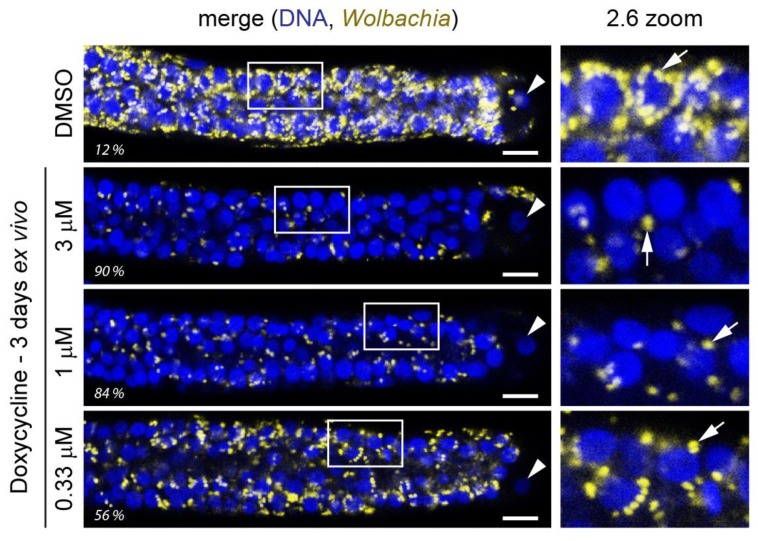
An ex vivo assay allows for rapid quantification of *Wolbachia* elimination in adult female worm ovaries near the distal tip cell due to compound treatment. Here, effects of a 3-day doxycycline treatment on female adult *B. pahangi* worms is shown. Worms are extracted from jirds and treated in 24-well plates, with one worm per well and usually 2 worms per treatment condition (with total of four ovaries analyzed). Worms are fixed, their ovaries dissected out, and stained with *Wolbachia*-specific 16S rRNA fluorescent in situ hybridization (FISH) (yellow). The stained ovaries are mounted on slides with DAPI-containing mounting medium to stain DNA (blue) and their distal ends imaged using a confocal microscope. The *Wolbachia*-specific 16S rRNA FISH is quantified by high content image analysis and normalized to DMSO control samples (percent elimination indicated here for each displayed ovary). Panels on the right are the enlarged sections demarcated with a white box in the ovary images. *Wolbachia w*Bp is indicated with arrows and the distal tip cell nucleus with an arrowhead. Scale bar = 10 µm.

Regardless, while validating *Wolbachia* elimination in filariae by any method is a significant first step in selecting attractive small molecules, being able to differentiate compound activities and select ones with the highest chance of success (rapid and total elimination of *Wolbachia*) is advantageous for efficient prioritization and development of superior chemical matter. Translation of validation results to in vivo efficacy has been variable, and likely not always related to absolute compound potency, but no consistent analysis comparing these methods has been undertaken thus far. Yet, in our experience the quantification of *Wolbachia* specifically near the distal tip cell of the ovaries of adult female *B. pahangi* worms following a 3-day treatment ex vivo (Figure 1) paired with optimization of exposures in vivo led to selection of exquisitely potent antiwolbachial compounds capable of eliminating >99% of *Wolbachia* from *L. sigmodonits* worms in vivo after a single dose [92]. Whether this indicates that elimination of *Wolbachia* found in the ovaries of filariae is relevant for sustained antiwolbachial effect, represents a proxy for efficient *Wolbachia* elimination throughout the worm, or if this population is particularly insensitive to some or all antiwolbachial treatments remains to be fully elucidated.

## 4. Promising Antiwolbachial Candidates

The cumulative effect of screening and validation activities has been the discovery and development of several promising antiwolbachial candidates. This includes novel and yet unoptimized chemical series, more advanced developed molecules, repurposing opportunities and alternative dosing regimens. The most advanced programs have molecules that are in or have undergone human trials, hoping to prove both safety and efficacy of these promising macrofilaricidal therapies.

### 4.1. Novel Chemical Series

**Figure 2:**
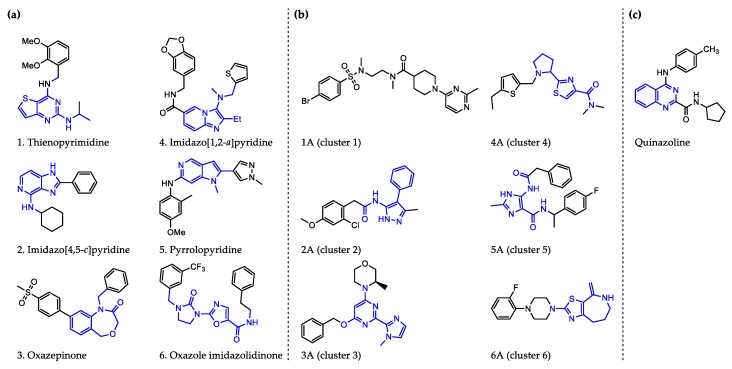
Representative structures of published antiwolbachial hit series identified in insect cell-based high-throughput screens with the cores of each series highlighted in blue. Series identified in screens using: (**a**) C6/36 insect cells with *w*Alb quantified using qPCR and Syto 11 high content imaging assay [87]; (**b**) C6/36 insect cells with *w*Alb quantified using immunofluorescence (*w*BmPAL antibody staining) [90]; (**c**) LDW1 insect cells with *w*Mel quantified using 16S rRNA FISH [92].

Perhaps some of the most intriguing leads in antiwolbachial drug discovery are small molecules with novel chemical structures. More than a dozen such series have been identified in screening efforts and published to date and represent a viable starting point for further medicinal chemistry optimization (Figure 2). Identified in phenotypic whole cell screens, the targets responsible for antiwolbachial activity of these novel compounds are currently unknown. However, focused chemical optimization efforts on the thienopyrimidine series [87] and the quinazoline series [92] have led to generation of molecules with excellent efficacy and properties: AWZ1066S and quinazolines CBR417 and CBR490 (Table 7).

#### 4.1.1. AWZ1066S

Chemical optimization of the thienopyrimidine series [87] identified in high-throughput screening led to the generation of AWZ1066, a compound with a quinazoline scaffold and increased efficacy against both insect and filarial *Wolbachia*. In the process of medicinal chemistry optimization over 300 analogs were synthesized and assessed for antiwolbachial activity and drug metabolism/pharmacokinetic (DMPK) properties [86]. AWZ1066 has two enantiomers, AWZ1066S and AWZ1066R, with AWZ1066S (Table 7) demonstrating slightly superior antiwolbachial activity. Assays used to assess activity of the compound during medicinal chemistry optimization included the 7-day in vitro C6/36 insect cells high content screening assay (SYTO 11) and a 6-day *B. malayi* microfilarial in vitro assay (qPCR), where both AWZ1066 enantiomers show activity superior to the doxycycline control. The good DMPK properties and potency of AWZ1066 translated well to in vivo models of filarial infection. When dosed for 7 days at 100 mg/kg *bid*, AWZ1066S eliminated 98% of *Wolbachia* from *B. malayi* adult female worms in SCID mice. The elimination was even greater, >99%, when AWZ1066S was similarly dosed for 14 days. Importantly, in the *L. sigmodontis* jird model of infection, a 7-day dosing regimen of 50 mg/kg *bid* eliminated >99% of *Wolbachia* in the adult female worms measured at 18 weeks post treatment, indicating elimination of bacteria to low enough titers to block repopulation of worm tissues after conclusion of treatment. This treatment also led to sterilization and gradual depletion of microfilariae from infected jird circulation. Together with favorable safety profiles, these data support AWZ1066S as an attractive novel antiwolbachial.

#### 4.1.2. Quinazolines CBR417 and CBR490

During the high-throughput screening using LDW1 insect cells and a high-content imaging assay relying on 16S rRNA FISH to detect *Wolbachia*, many novel small molecules and known drugs were identified as potent against *w*Mel strain of *Wolbachia*. However, novel compounds with a quinazoline scaffold had the additional advantage of being active against filarial *Wolbachia* found in adult *B. pahangi* ovaries in a 3-day ex vivo assay at levels superior to the doxycycline control. Focusing on DMPK properties and potency against filarial *Wolbachia* found in adult female *B. pahangi* ovaries, medicinal chemistry optimization of the quinazoline series generated quinazolines CBR417 and CBR490 (Table 7), with improved potencies compared to doxycycline in both the in vitro and ex vivo assays, excellent exposure and safety profiles. This translated to unparalleled in vivo efficacy: in the mouse model of *L. sigmodontis* infection a single dose of either quinazoline at 200 mg/kg caused a >99% elimination of *Wolbachia* from adult female worms. More modest doses given over longer periods of time caused an equivalent *Wolbachia* reduction (e.g., 2 doses of 100 mg/kg given once per week). Additionally, a less advanced quinazoline analog from the series, CBR715, showed strong efficacy in adult *B. malayi* females and *O. ochengi* males in SCID mouse models of infection, causing >99% *Wolbachia* elimination when dosed at 50 mg/kg *bid* for 7 days. Dose range finding studies would need to be completed in these models to determine whether further dose and time reductions of treatment would be possible with CBR417 and CBR490. Nonetheless, these preliminary data suggest that the series has excellent potential to be developed as significantly abbreviated antiwolbachial therapies. Finally, the conspicuous structural similarity of CBR417 and CBR490 to the independently-developed AWZ1066S may suggest a related mode of action for these novel molecules. However, quinazoline heterocycles are present in many biologically active compounds found throughout drug discovery. Therefore, whether these molecules have the same or similar targets relevant for their antiwolbachial effect remains to be determined. 

### 4.2. Repurposing of Known Drugs and Alternative Dosing Regimens

#### 4.2.1. Minocycline

Minocycline is a tetracycline class antibiotic related to doxycycline with antiwolbachial activity observed in insect cells and in filarial nematodes [40,88,92]. In filarial nematodes in vitro [40], ex vivo [92] and in vivo [88], minocycline activity is superior compared to equivalent doses of doxycycline Pharmacodynamic modeling also suggests the superiority of minocycline over doxycycline when dosed proportionally [99]. A recent human pilot trial compared the efficacy of a 4-week or 3-week 200 mg/day doxycycline to that of a 3-week 200 mg/day minocycline therapy in *O. volvulus* infected individuals in Ghana [104]. While the findings were not conclusive due to the small size of the study, a trend of increased potency of minocycline was observed compared to the 3-week 200 mg/day doxycycline, but with the 4-week doxycycline treatment ultimately showing superior efficacy. Therefore, more comprehensive studies are required to assess the possibility of minoncycline as a faster-acting antiwolbachial therapy. Methacycline, an older tetracycline antibiotic, was also shown to have superior activity compared to doxycycline in vivo [88] but has not garnered the same attention as minocycline likely because of its lower absorption and lipophilicity compared to either doxycycline or minocycline [105]. 

#### 4.2.2. High Dose Rifampicin

Rifampicin is a clinically approved antibiotic used in the treatment of tuberculosis that inhibits RNA synthesis by binding to and inhibiting the DNA-dependent RNA polymerase of bacteria. The long-standing interest in rifampicin as an antiwolbachial [80] has resulted in many studies of its activity and it’s potential as a macrofilaricide has been thoroughly explored [40,77,106]. Rifampicin is very potent in vitro against *Wolbachia* in insect cells and demonstrates in vivo efficacy in rodent animal models when dosed appropriately. Additionally, it is less toxic than doxycycline and can be administered to children. However, in in vivo models and clinical trials using standard dose of rifampicin approved for tuberculosis treatment (10 mg/kg), rifampicin failed to show the anticipated efficacy. For example, in a clinical trial its ability to clear *Wolbachia* from *O. volvulus* when given at 10 mg/kg over 2 or 4 weeks to infected patients was shown to be inferior to that of a 6-week 200 mg/kg doxycycline treatment [107]. Additionally, in *W. bancrofti* patients, a 2-week long combination treatment of 200 mg/kg doxycycline plus 10 mg/kg rifampicin was also inferior to a 4-week 200 mg/kg doxycycline treatment [65]. Similar results were observed in cattle infected with *O. ochengi* [102]. However, recent investigations into tolerability of higher dose of rifampicin for tuberculosis treatments combined with lower costs of synthesis, prompted further investigations into efficacious dose modeling for rifampicin as an antiwolbachial [108]. Results from in vivo dose escalation studies and drug exposure modeling simulations suggest that a minimum dose of 30–40 mg/kg in humans could achieve rifampicin exposures conducive to elimination of *Wolbachia* and a macrofilaricidal effect. Based on in vivo efficacy studies in SCID mouse models of *B. malayi* and *O. ochengi* infection, the high dose rifampicin treatment would likely need to be administered for at least one week to treat lymphatic filariasis and two weeks to treat onchocerciasis. The shortened dosing regimens have an added advantage in that they may be less likely to cause development of antibiotic resistance in tuberculosis patients. 

#### 4.2.3. Corallopyronin A

Corallopyronin A is a natural product of *Corallococcus coralloides* B035 and a noncompetitive inhibitor of bacterial DNA-dependent RNA polymerase. It inhibits the RNA polymerase via a mechanism different from rifampicin and has low efficacy against *Mycobacterium spp.*, suggesting that unlike rifampicin, it could be administered without the concern over development of antibiotic resistance in tuberculosis patients, inciting investigations into its antiwolbachial activity [109,110]. In fact, corallopyronin A was shown to be effective against *Wolbachia in vitro* and in vivo but due to limitation in the amount of corallopyronin A that was available at the time the in vivo studies were conducted, superiority to doxycycline has thus far not been determined. Nonetheless, corallopyronin A may be an attractive antiwolbachial alone or in combination, especially when considering the development of antibiotic resistance due to antiwolbachial antibiotic treatment in patients co-infected with *Mycobacterium tuberculosis*.

#### 4.2.4. DNA Gyrase Inhibitors: Fluoroquinolones and Aminocoumarins

Fluoroquinolones possess variable antiwolbachial activity. Ciprofloxacin, initially tested in Aa23 *w*AlbB-infected cells showed no [80] or moderate effect (MIC = 2–4 µg/mL) [77] on *Wolbachia* titers. Ofloxacin and levofloxacin that were also tested in vitro showed similarly moderate effects [77]. Initial *L. sigmodontis in vivo* studies with ciprofloxacin also resulted in disappointing results [111]. Further work led to re-discovery of fluoroquinolones as potential antiwolbachials with ciprofloxacin, moxifloxacin and sparfloxacin demonstrating activities in vitro in C6/36 insect cells [88]. In the same study, ciprofloxacin and moxifloxacin also showed antiwolbachial effects in an adult *O. gutturosa* in vitro assay, and ciprofloxacin and sparfloxacin showed reduction in *Wolbachia* loads and reductions in *L. sigmodontis* worm length in vivo when dosed one day following initiation of filarial infection for 14 days. More recently, other fluoroquinolones were identified as having activity against *w*Mel *Wolbachia* in LDW1 cells (delafloxacin, finafloxacin, nadifloxacin, sitafloxacin) with EC_50_ values ranging between 243 nM and 808 nM [92]. However, they were not active against *w*Bp in adult *B. pahangi* females in a 3-day ex vivo assay, causing at most a 25% reduction in *Wolbachia* in worm ovaries following a 1 µM treatment. Interestingly, other antibiotics targeting the bacterial DNA gyrase, the aminocoumarins, showed similar patterns of activity in the same study. Aminocoumarins were exquisitely potent against *w*Mel in insect cells (coumermycin EC_50_ = 1.5 nM) but were not able to clear *Wolbachia* effectively from ovaries of *B. pahangi* (44% *w*Bp elimination at 1 µM coumermycin). This may reflect the lower potency of both fluoroquinolones and aminocoumarins against filarial *Wolbachia*, their slower mode of action, or insufficient accumulation in worm tissues. Therefore, identification or synthesis of more effective analogs would likely be required for these compounds to be deployed as macrofilaricidal therapies. 

#### 4.2.5. Kirromycins

Kirromycin, also known as mocimycin, is an elfamcyin class antibiotic with a narrow spectrum of activity. Kirromycin inhibits protein synthesis by interacting with the prokaryotic elongation factor Tu (EF-Tu), trapping EF-Tu on the ribosome and preventing the next round of elongation [112]. Recently, kirromycin B was identified as a potent antiwolbachial in a screen of the Natural Products Library (NPL) constructed by the The Natural Products Library Initiative at the Scripps Research Institute. The NPL consists of crude microbial extracts, fractions generated by medium-pressure liquid chromatography (MPLC), and pure natural products [93]. Kirromcyin B showed excellent antiwolbachial activity in vitro in *Drosophila* insect cells (IC_50_ = 0.58 nM) and ex vivo in *B. pahangi* adult worm ovaries (90% *Wolbachia w*Bp elimination from ovaries of worms treated for 3 days with 1 µM kirromycin B). Investigation of the kirromycin B-producing strain *Streptomyces* sp. CB00686 led to identification of two additional congeners, kirromycin and kirromycin C as having similar antiwolbachial activities in vitro (IC_50_s of 0.25 nM and 1.08 nM, respectively) and slightly lower activities ex vivo (65% and 67% elimination at 1 µM, respectively). Interestingly, similar levels of activity were observed ex vivo at lower concentrations for all three compounds (0.33 and 0.11 µM) as at 1 µM, suggesting perhaps a potent but bacteriostatic mode of action (a similarly “flat”, albeit less-potent, antiwolbachial activity profile was observed for Tylosin A [92]). While these data are very promising, investigation in in vivo models of filarial infection are required to determine the utility of kirromycins and other elfamycins as antiwolbachial candidates for treatment of filarial worm infections.

### 4.3. Chemically-Optimized Antibiotics

#### 4.3.1. Boron-Pleuromutilin, AN11251

**Figure 3:**
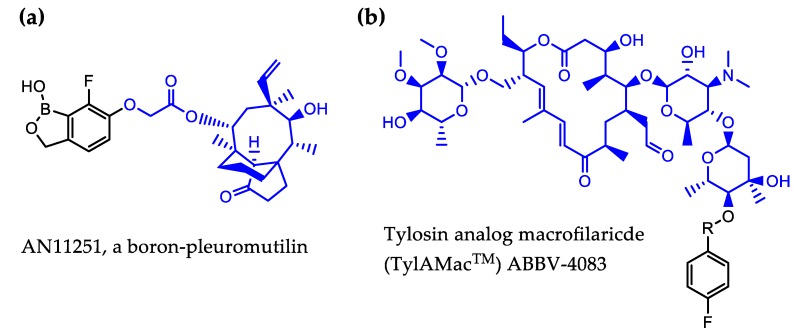
Structures of AN11251 (**a**) and the tylosin core of ABBV-4083 (TylAMac) (**b**). The antibiotic core of each molecule is colored blue.

Benzoxaboroles have been found to impart favorable properties to existing drug scaffolds and boron-containing chemistry has been successfully used by Anacor Pharmaceuticals (recently acquired by Pfeizer) to generate potent molecules for several infectious disease indications. In addition to FDA-approved anti-fungal therapy, this includes the modification of pleuromutilin class of antibiotics, shown to have antiwolbachial activity in vitro [85]. Pleuromutilins are bacteriostatic protein synthesis inhibitors that act by direct binding to the peptidyl-transfer center of the bacterial ribosome. Pleuromutilin itself does not possess strong antiwolbachial activity (*w*AlbB EC_50_ > 1 µM), with other pleuromutilins more potent against *Wolbachia* in insect cells [85,92]. For example, valnemulin in LDW1 cells has EC_50_s ranging between 6.1–30 nM and is active against *Wolbachia* in *B. pahangi* ovaries as assessed in a short 3-day ex vivo assay where 1 µM valnemulin treatment causes 67% *w*Bp elimination from ovaries, activity that is similar to that of doxycycline [85,92]. Using boron-containing chemistry to impart more favorable properties to pleuromutilins, boron-pleuromutilin series optimization led to the generation of AN11251 (Figure 3(a)), which was exquisitely potent in vitro against C6/36 *Wolbachia*-containing insect cells (*w*Alb EC_50_ = 15 nM). The activity of AN11251 was confirmed in vivo in the *L. sigmodontis* mouse model of infection, where oral administration of AN11251 at 50 mg/kg *bid* for 14 days caused a >99% elimination of *Wolbachia* from adult female worms. However, dosing could not be reduced sufficiently to meet the < 7-day dosing target. Therefore, further investigations to determine if AN11251 dosing regimen can be abbreviated by combination therapy are underway.

#### 4.3.2. Tylosin Analog ABBV-4083

Tylosin is a macrolide antibiotic and an animal food additive used in agriculture. Like pleuromutilins, tylosins are bacteriostatic and inhibit protein synthesis by binding to the bacterial ribosome, specifically in the peptide exit tunnel of the bacterial 50S ribosomal subunit [113]. Activity against *Wolbachia* discovered in insect cells prompted the chemistry optimization program led by AbbVie together with the A-WOL Consortium to increase the oral bioavailability of tylosin and generate a tylosin analog microfilaricide (TylAMac™) [114,115]. The generated lead molecule ABBV-4083 (Figure 3b) showed a potent in vitro antiwolbachial activity in insect cells (EC_50_ = 0.019 nM) that was nearly 1500-fold more potent than that of Tylosin A [114]. ABBV-4083 also demonstrated in vivo efficacy and superiority over doxycycline in a jird model of infection, where a 14-day treatment with 150 mg/kg PO ABBV-4083 resulted in a 99.91% *Wolbachia* elimination as measured 16 weeks post-treatment initiation, blocked embryogenesis and a complete clearance of circulating microfilariae [114]. ABBV-4083 performed well in preclinical safety studies and showed further efficacy in additional in vivo models of filarial infection [115]. Furthermore, based on its relatively low anti-*L. loa* mf in vitro activity (IC_50_ = 23.3 µM) it was predicted to be safe to administer to patients co-infected with *L. loa* [115]. In 2018 in partnership with the Liverpool School of Tropical Medicine and the Drugs for Neglected Diseases initiative (DND*i*), Abbvie successfully completed a phase I clinical trial assessing the safety and tolerability of ABBV-4083. The encouraging findings from this trial support advancement of ABBV-4083 to phase II clinical trials (https://www.dndi.org/diseases-projects/portfolio/abbv-4083/).

### 4.4. Combination Therapies and Target-Based Screening

What are some of the other directions for future antiwolbachial macrofilaricide development? In addition to continued optimization and preclinical development of novel small molecule candidates using recently validated workflows, profiling of efficacy of combination therapies is an attractive strategy. The recognized challenge of developing effective fast-acting compounds may be overcome by pairing antiwolbachials with synergistic activities or with other antifilarial compounds such as albendazole to deliver superior treatments. This approach is not always successful: pairing of doxycycline with rifampicin does not improve outcomes and may even lead to inferior results. However, examples highlighting the potential of this strategy include in vivo studies pairing albendazole with minocycline or rifampicin [116], and other combination antibiotic treatments (e.g., rifapentine and moxifloxacin) [106], all showing superior efficacy of combinations versus monotherapies. A small pilot trial has also demonstrated that combining albendazole with doxycycline may provide a shorter treatment course [104]. The success of albendazole as a synergistic partner with other antiwolbachial therapies may stem from its action as a filarial microtubule inhibitor with indirect effects on *Wolbachia*. Regardless, albendazole’s purported antifilarial effect and extensive use in patients infected with filaria (including those with loiasis) makes albendazole an attractive partner for antiwolbachial therapy. 

High-throughput and more focused screening that identified antibiotics with known mode of action has also indirectly identified druggable *Wolbachia* targets that it shares with other bacterial pathogens. This includes the ribosome (tetracyclines, pleuromutilins, macrolides), the DNA-dependent RNA polymerase (rifamycins, corallopyronin A), the DNA gyrase (fluoroquinolones, aminocoumarins), lipoprotein signal peptidase II (LspA) (globomycin) [84] and finally the peptide deformylase, as a number of peptide deformylase inhibitors have recently been identified as having antiwolbachial activity [92]. However, target-based screening has not enjoyed the same success and prevalence as phenotypic screens in antiwolbachial drug discovery largely due to lack of validated druggable *Wolbachia* targets outside this standard antibacterial repertoire. The few efforts made in this arena include biochemical screens identifying inhibitors of the *B. malayi* FtsZ GTPase [117] and inhibitors of heme pathway targeting the *Wolbachia* δ-aminolevulinic acid dehydratase (wALAD) [118,119]. Yet as targets of novel validated antiwolbachial compounds are identified, target-based screening may take a more prominent role in antiwolbachial drug discovery efforts in the future.

## 5. Perspectives for the Future of Antiwolbachial Drug Discovery

Over the last two decades, significant strides were made towards the discovery of antiwolbachial therapies for filarial worm infections. Through the support of charitable organizations such as the Bill and Melinda Gates Foundation, the Global Health Innovative Technology Fund (GHIT), DND*i* and other funding bodies, the organized and targeted efforts of the A-WOL Consortium [120] led by the Liverpool School of Tropical Medicine in collaboration with other academic and industrial partners (Abbvie, Anacor, AstraZeneca, Eisai, New England Biolabs, University of Buea, University of Bonn, University of Liverpool and others), many novel chemical structures with in vitro antiwolbachial activity have been identified and even advanced through clinical studies. This is largely owing to the successful high-throughput screening and validation frameworks that have been developed and yielded a wealth of results in the last five years. However, compared with other neglected tropical diseases, the clinical development pipeline for macrofilaricides remains scarcely populated. Following favorable phase I results, the tylosin analog ABBV-4083 will likely progress into Phase II clinical trials. Clearly, much depends on the success of this clinical candidate and its ability to contribute to filarial disease elimination goals. Securing the significant funding required for pre-clinical and clinical development of novel small molecules may be both challenging yet instrumental in the event of failure or uncovered liabilities of candidates in advanced studies. The lessons learned over the last five years of intensive antiwolbachial drug discovery should prove vital in the event of unfavorable results.

## Figures and Tables

**Table 1:** Overview of parasitic filarial nematodes pertinent to the antiwolbachial approach.

Filarial Nematode	Common Host	*Wolbachia*	Vector	Disease	Location in Host	General Symptoms	Geographical Distribution	Ref.
*Onchocerca volvulus*	humans	yes	black flies, *Simulium* spp.	onchocerciasis, aka. river blindness	adults in subcutaneous nodules, mf migrate through skin and eyes	skin disease (onchodermatitis: itching, depigmentation), onchocercomata (nodules), blindness, neurological disease (nodding syndrome, Nakalanga syndrome, epilepsy)	sub-Saharan Africa, small foci in South America and Yemen	[1,2]
*Onchocerca ochengi*	cattle (experimental models: mice)	yes	black flies, *Simulium* spp.	onchocerciasis aka. onchocercosis	adults in intradermal nodules, occasionally in subcutis	intradermal nodules (noted due to damage to bovine hides); other clinical impact unknown	documented in Uganda and Cameroon; used as a model to study filarial infection	[3,4]
*Wuchereria bancrofti*	humans (~90% of LF cases)	yes	mosquitoes	lymphatic filariasis, aka. Bancroftian filariasis	adults in lymphatic vessels; mf in peripheral blood with varying periodicities	mostly asymptomatic but with time cause damage to lymphatic system and kidneys; chronic symptoms include lymphoedema, elephantiasis, hydroceles;acute symptoms include local inflammation, fevers, secondary bacterial infections, acute filarial lymphangitis, acute dermatolymphangioadenitis	tropics in Asia, Africa, Pacific, and Americas	[1,5,6,7,8,9,10]
*Brugia malayi*	humans (experimental models: mice, jirds)	yes		lymphatic filariasis, aka. Brugian filariasis			East and South Asia	
*Brugia timori*	humans	yes					Indonesia and Timor-Leste	
*Brugia pahangi*	cats, dogs, rarely humans (experimental models: jirds)	yes					Malaysia, Thailand, and Indonesia	
*Mansonella ozzardi*	humans	yes	biting midges (mostly *Culicoides*) and black flies, *Simulium* spp.	ozzardi mansonellosis	uncertain; adults potentially in subcutaneous tissues/thoracic and peritoneal cavity; mf in blood and skin	potential ocular lesions; mostly asymptomatic but also fever, headaches, itching, joint pain, rash, sensation of coldness in the legs, foot and face edema, keratitis	Caribbean, the Amazon, border between Bolivia and Argentina	[11,12]
*Mansonella perstans*	humans and primates	yes (potentially strain dependent)	biting midges (*Culicoides*)	mansonellosis	adults in serous body cavities, may also appear subcutaneously; mf in blood	mostly asymptomatic; occasionally Calabar swellings, itching, pruritus, joint pain, enlarged lymph glands, neurological symptoms	western, eastern, central Africa; equatorial Brazil to Caribbean	[13,14]
*Mansonella streptocerca*	humans and primates	not reported	biting midges (*Culicoides*)	mansonellosis	adults in subcutaneous tissues; mf in skin	mostly asymptomatic; occasionally dermatitis, pruritus, rash, papular skin, inguinal adenopathy, dizziness	western, eastern, central Africa	[13]
*Litomosoides sigmodontis* (aka. *Litomosoides carinii* in older literature)	cotton rats *Sigmodon hispidus* (experimental models: rats, *Mastomys*, mice, jirds)	yes	rat mites (*Ornithonyssus bacoti*)	cotton-rat filariasis	adults in pleural cavity (less commonly in peritoneal cavity); mf in peripheral blood	can cause wasting and affect survival; pathological changes in lungs, spleen and lymphatics; scattered myocarditis	likely southeastern United States, Mexico, and Central America; used as a model to study filarial infection	[15,16,17,18,19]
*Dirofilaria immitis*	companion animals (mainly dogs but also cats, ferrets) and wild animals (wolves, coyotes, foxes, pinnipeds, raccoons, etc.); can also infect humans with *D. repens* infecting humans to a greater extent than *D. immitis*	yes	mosquitoes	dirofilariasis/ dirofilariosis, aka. heartworm disease	heart and pulmonary arteries	in dogs: cough, exercise intolerance, fainting, coughing up blood, severe weight loss, congestive heart failure	most countries with temperate, semitropical or tropical climates	[20,21]
*Dirofilaria repens*				subcutaneous dirofilariasis/ dirofilariosis	adults in subcutaneous tissues; mf in peripheral bloodstream	mostly asymptomatic; occasionally pruritus, dermal swelling, subcutaneous nodules containing the parasite, and ocular conjunctivitis	Europe, Asia, Africa	[22]
*Loa loa^b^*	humans (experimental models: primates (e.g., baboons), rodents)	no	deerflies, genus *Chrysops*	loiasis, aka. African eye worm	connective tissue	mostly asymptomatic, eye worm, Calabar swellings, itching, tiredness, muscle and joint pain, hives	West and Central Africa	[23]

* in specified host; LF, lymphatic filariasis; mf, microfilariae.

**Table 2:** Antifilarial parasitic drugs.

Ivermectin	Diethylcarbamazine (DEC)	Albendazole
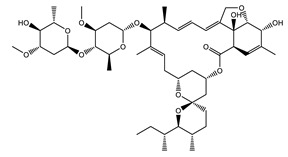 macrocyclic lactone	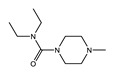 piperazine derivative	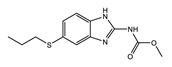 benzimidazole
MOA not fully understood; causes paralysis by binding to glutamate-gated chloride channels of parasitic worms affecting their motility, feeding, and reproduction	MOA not fully understood; inhibitor of arachidonic acid metabolism in microfilariae and host; dependent on host iNOS; likely a host innate immunity mediated effect	Blocks parasite microtububle assembly (binds to β-tubulin); most active against intestinal parasites

MOA, mechanism of action.

**Table 3:** Guidelines for treatment of filarial parasite infections through preventive chemotherapy mass drug administration campaigns.

Disease	Areas not Co-endemic with Onchocerciasis	Areas Co-endemic with Onchocerciasis	Areas Co-endemic with Loiasis
Onchocer-ciasis	–	ivermectin (150–200 µg/kg)	not advised; test and not treat strategies investigated
Lymphatic filariasis	once a year DEC (6 mg/kg) and albendazole (400 mg); *2018–2019 start triple therapy in select countries*	once a year ivermectin (200 µg/kg) with albendazole (400 mg)	twice a year albendazle (400 mg)
Loiasis	DEC or albendazole; treatment not always recommended due to risk of SAEs; *no programs currently in place to control or eliminate loiasis*		

DEC, diethylcarbamazine citrate; SAEs, severe adverse events.

**Table 4:** *Wolbachia* strains of filarial nematodes of clinical relevance and ones used for antiwolbachial drug discovery.

Filarial Nematode	Significance	*Wolbachia*			
		Strain	Super-group	Genome Size (Mb) *	Proteins *
*Onchocerca volvulus*	clinical	*w*Ov	C	0.96	649
*Onchocerca ochengi*	advanced screen for drug and vaccine development [3,4]	*w*Oo	C	0.96	651
*Onchocerca gutturosa*	in vitro screen for drug development [40,41]	*w*Og	C	–	–
*Dirofilaria immitis*	dog heartworm (veterinary) [42]	*w*Dim	C	0.92	823
*Wuchereria bancrofti*	clinical	*w*Wb	D	1.06 (*draft*)	961 (*draft*)
*Brugia malayi*	clinical; rodent efficacy model for drug and vaccine development [4]	*w*Bm	D	1.08	839
*Brugia timori*	clinical	*w*Bt	D	–	–
*Brugia pahangi*	rodent efficacy model for drug and vaccine development [17]	*w*Bp	D	1.4 (*draft*)	803 (*draft*)
*Litomosoides sigmodontis*	rodent efficacy model for drug and vaccine development [17,18,43,44,45]	*w*Ls	D	Data available, but not yet published **	
*Loa loa*	clinical; microfilarial counter-screen for drug development [46]	–	–	–	–

* Genome size and protein number were taken from the NCBI Genomes database for *Wolbachia w*Ov [47] (BioProject PRJEB4840), *w*Oo [48] (BioProject PRJEA81837), *w*Wb [49] (BioProjects PRJNA388334), *w*Bm [50] (BioProjects PRJNA12475), and *w*Dim [42] and *w*Bp [51] as published. ** http://nematodes.org/genomes/litomosoides_sigmodontis/

**Table 5:** Insect cells used for antiwolbachial high-throughput screening and features of *Wolbachia* strains therein.

Cell Line			*Wolbachia*				References
Cell Line	Species	Markers	Strain	Super-group	Genome Size (Mb)	Proteins	
Aa23	*Ae. albopictus*	–	*w*Alb	B	1.48	1205	[77,78,79,80]
C6/36	*Ae. albopictus*	–					[81,82,83,84,85,86,87,88,89,90]
JW18	*D. melanogaster*	Jupiter-GFP	*w*Mel	A	1.27	1100	[71,72,91]
LDW1	*D. melanogaster*	Jupiter-GFP, Histone-RFP					[72,75,85,92,93]

**Table 6:** *Wolbachia* visualization and quantification methods and their utility in high-throughput screening.

Quantification Method	Advantages	Disadvantages	Applied to HTS?
Giemsa	simple, inexpensive	non-specific	no
Propidium iodide	simple, inexpensive	non-specific	no
DAPI	simple, inexpensive	non-specific	384-well [71]
Syto 11	simple, moderately priced	non-specific	384-well [89]
qPCR	*Wolbachia* specific	complex, higher expense	96-well [87,88]
qRT-PCR	*Wolbachia* specific	much more complex, higher expense	no
Immuno-fluorescence	*Wolbachia* specific, simple	higher expense, relies on limited reagent (anti-*Wolbachia* antibody), more complex than one-reagent protocols (e.g., Syto 11)	384-well [90]
16S rRNA FISH	*Wolbachia* specific, simple, inexpensive customizable probes	more complex than one-reagent protocols (e.g., Syto 11)	1536-well [92,93]

HTS, high-throughput screening.

**Table 7:** Advanced novel small molecules optimized for antiwolbachial efficacy.

	AWZ1066S [86]	CBR490 [92]	CBR417 [92]
Series	azaquinazoline	quinazoline (methylpyridine)	quinazoline (oxadiazole)
Structure	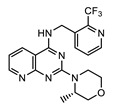	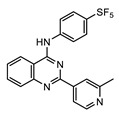	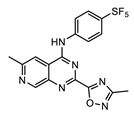
Activity	C6/36 EC_50_ = 2.5 nM; microfilariae EC_50_ = 121 nM;	LDW1 EC_50_ = 33 nM; *B. pahangi* ovaries ex vivo EC_50_ < 111 nM	LDW1 EC_50_ = 24 nM; *B. pahangi* ovaries ex vivo EC_50_ = 356 nM
Efficacy	>99% *w*LS depletion in adult female *L. sigmodontis:* 50 mg/kg *bid* 7 days >90% *w*Bm depletion in adult female *B. malayi:* 100 mg/kg *bid* 7 days	>99% *w*LS depletion in adult female *L. sigmodontis:* 200 mg/kg SINGLE DOSE, or 100 mg/kg one dose given per week ×2, or 30 mg/kg *qd* (*quaque die*, once a day) for 7 days	>99% *w*LS depletion in adult female *L. sigmodontis:* 200 mg/kg SINGLE DOSE, or 100 mg/kg one dose given per week x2, or 60 mg/kg *qd* for 4 days
